# Low-Bit Rate Feedback Strategies for Iterative IA-Precoded MIMO-OFDM-Based Systems

**DOI:** 10.1155/2014/619454

**Published:** 2014-02-11

**Authors:** Sara Teodoro, Adão Silva, Rui Dinis, Atílio Gameiro

**Affiliations:** ^1^DETI, Instituto de Telecomunicações, Universidade de Aveiro, Campus Universitário de Santiago, 3810-193 Aveiro, Portugal; ^2^Instituto de Telecomunicações, Faculdade de Ciências e Tecnologia, Universidade Nova de Lisboa, 2829-516 Caparica, Portugal

## Abstract

Interference alignment (IA) is a promising technique that allows high-capacity gains in interference channels, but which requires the knowledge of the channel state information (CSI) for all the system links. We design low-complexity and low-bit rate feedback strategies where a quantized version of some CSI parameters is fed back from the user terminal (UT) to the base station (BS), which shares it with the other BSs through a limited-capacity backhaul network. This information is then used by BSs to perform the overall IA design. With the proposed strategies, we only need to send part of the CSI information, and this can even be sent only once for a set of data blocks transmitted over time-varying channels. These strategies are applied to iterative MMSE-based IA techniques for the downlink of broadband wireless OFDM systems with limited feedback. A new robust iterative IA technique, where channel quantization errors are taken into account in IA design, is also proposed and evaluated. With our proposed strategies, we need a small number of quantization bits to transmit and share the CSI, when comparing with the techniques used in previous works, while allowing performance close to the one obtained with perfect channel knowledge.

## 1. Introduction 

Coordination between cells is one of the fastest growing topics of research in wireless communications, and it is a promising solution for cellular wireless systems to mitigate intercell interference and provide the increased capacity expected in the forthcoming years [1, 2]. This technology is already under study in long-term evolution advanced (LTE A) under the coordinated multipoint concept (CoMP) [3]. Since the performance of cell-edge users is greatly limited by the intercell interference, the design of an efficient interference management scheme is crucial to improve the performance of those users.

One interesting recent scheme to efficiently eliminate the intercell interference and achieve a linear capacity scaling (i.e., the sum rates increase linearly with the number of users at high SNR) is interference alignment (IA) [4]. IA was firstly introduced for MIMO X channel in [4] and subsequently in the context of the *K*-user interference channel in [5]. With this technique, the transmitters align in the unwanted users' receive signals in a subspace orthogonal to the subspace used for that users' data, through the use of appropriate precoders and thus allowing to achieve the maximum degrees of freedom (DoF). It was shown in [5] that the capacity of an interference channel (IC) is for a given user one-half the rate of its interference-free capacity in the high transmit power regime, regardless of the number of users.

A closed-form solution for constant channels is still unknown for more than 3 users. An explicit formulation of the precoding vectors achieving IA for time or frequency selectivity channels was presented in [5]. A two-stage optimization of the precoding and decoding matrices in the *K*-multi-input, multioutput (MIMO) constant interference channels was proposed in [6]. Some iterative algorithms were also proposed for these multiuser MIMO systems [7–9]. In [7], the Max-SINR algorithm was proposed, where instead of minimizing interference power at each iteration, the algorithm iteratively maximizes the per-stream signal interference-plus noise ratio (SINR). A minimum mean squared error- (MMSE-) based iterative IA scheme has been proposed in [8], by relaxing the need for perfect alignment while minimizing the signal's sum mean square error. A comparison between several iterative linear precoding designs using alternating minimization was performed in [9].

The knowledge of CSI at the transmitter is absolutely crucial for the precoder-based systems. When perfect channel state information (CSI) is available, IA achieves the optimum theoretical bound of DoF for interference channels [4]. However, assuming perfect CSI at the transmitters is not realistic in most of the scenarios, and the requirement of accurate CSI knowledge in all the cooperating nodes incurs in a large overhead penalty. Some works present the IA technique to improve a user communication in a cell-edge scenario for cellular networks using a leakage-based strategy, where only the interference from the stronger base station (BS) is cancelled. This decreases the CSI overhead since it only needs to perfectly know the CSI of one interferer, though this approach may not be realistic for some scenarios, where more than one relevant interferer does exist [10, 11].

Some studies addressed the issue of CSI knowledge in the IA context through channel reciprocity, as in [12], where several iterative algorithms take advantage of the reciprocity of wireless networks to achieve IA. In such cases, IA precoders are generated through an iterative procedure consisted in sending pilots from the transmitters and estimating interference covariance matrices at the receivers [12]. However, this method relies on pilots transmissions, which incurs in a nonnegligible overhead. Also this method cannot be used in frequency-division duplexed- (FDD-) based systems and requires tight synchronization in time-duplexed systems [13, 14].

Another strategy to solve the CSI knowledge at the transmitters is to feed back the channels from user terminals (UTs) to the BSs [15–17]. Limited feedback, where the CSI is quantized and fed back to the transmitter through a limited link, was first introduced in [18], to single antenna systems using efficient quantization via what is known as Grassmannian codebooks. Nevertheless, the complexity of quantized feedback increases with codebook size and large Grassmannian codebooks are difficult to design and encode. Moreover, it cannot be applied to the systems where the CSI exhibits no special structure, as the main case of interest of IA in multiple antenna systems, where the CSI to be fed back is a set of channel matrices [13]. In [15], the theoretical boundary of DoF achieved with a limited feedback for MIMO multi-user systems is derived and an appropriate scheme is proposed. Random vector quantization (RVQ) codebooks are used in these cases because the optimal vector quantizer for this problem is not known in general [16, 17]. Although RVQ techniques allow efficient IA schemes with limited feedback, the required codebooks can be very large, especially when we have a high number of transmit and receive antennas. Moreover, while RVQ performs well when there are a large number of mobiles relative to the number of transmit antennas, it performs poorly in the small system regime [19]. As a solution to this problem, quantization codebook size must scale exponentially with SNR, having however the drawback of becoming extensively large for high SNR. The encoding and decoding complexity can be very high, even for flat fading channels, becoming prohibitively high for severely time-dispersive channels. In that case, it is preferable to employ simpler quantizers, working on a sample-by-sample basis, as in [20].

To overcome the problem of scaling complexity (codebooks and computation complexity to quantize the CSI increases with the number of users and antennas), IA with analog feedback was considered in [14], where the channel coefficients are directly transmitted as uncoded quadrature and amplitude modulated symbols. However, analog feedback does not warrant an accurate CSI and consequently a full multiplexing gain, if the forward and reverse link SNRs do not scale together (i.e., both SNRs increase together, so that when the SNR on the reverse link is high we also have high-quality CSI). Thus, the development of new feedback strategies with reduced overhead applicable with no channel restrictions is crucial to achieve the maximum DoF.

In this paper, we design new quantization strategies that allow efficient CSI feedback for IA-precoder MIMO-OFDM-based systems. A quantized version of the CSI associated with the different links between BS and UT is fed back from the UT to the BS and sent to the other BSs through a limited-capacity backhaul network. The proposed quantization strategies have low-complexity and low-bit rates just quantizing part of the samples of either the channel frequency response (CFR) or the channel impulse response (CIR). These quantized channels are then employed by the different BSs to perform the overall IA design. Our channel quantization methods have much lower complexity than the RVQ-based techniques, since they do not require the use of large codebooks. Moreover, for severely time-dispersive channels, the RVQ-based schemes require the quantization of all subchannel samples; thus, the proposed scheme is even more advantageous for these cases, with lower feedback overhead. As the iterative IA MMSE-based algorithms have some degradation in high SNR due to channel errors, a new robust iterative IA technique is also proposed and evaluated using the proposed feedback strategies, where channel quantization errors are taken into account in IA design.

The remainder of the paper is organized as follows.  Section 2 presents the system model of the *K*-user IC MIMO for OFDM systems. The proposed channel feedback strategies are presented in Section 3.  Section 4 briefly describes the proposed IA algorithms to be evaluated with limited feedback.  Section 5 presents the main simulation results. The conclusions and future work are drawn in Section 6.

*Notation.* Boldface capital letters denote matrices; boldface lowercase letters denote column vectors. The operation (·)^*H*^ represents the Hermitian transpose of a matrix; *Re*(·) and *Im*⁡(·) represent the real and imaginary parts of a complex number, respectively; and arg(·) representes the phase of a complex number. E[·] stands for the expectation; ||·|| represents the Frobenius norm of a matrix; *ι* is the imaginary unit; and, tr⁡(·) refers to the trace of a square matrix.

## 2. System Characterization

**Figure 1:**
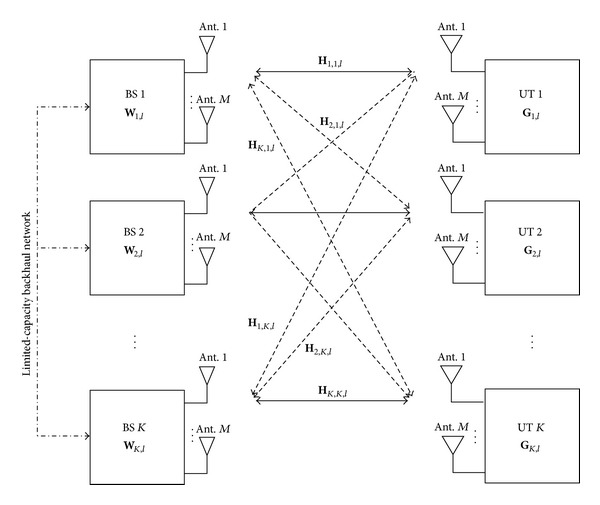
A generic block diagram of the considered scenario.

In this paper, we consider the downlink of a multicell MIMO-OFDM-based system. Due to the orthogonality between subcarriers, we can apply IA independently for each OFDM subcarrier. Therefore, we have a *K*-user MIMO interference channel with constant coefficients on a per-subcarrier basis. It comprises a *K* transmitter-receiver pair sharing the physical channel, with a given transmitter intending to have its signal decoded only by a single receiver. In a downlink-cellular-based system, the transmitter and receiver correspond to the BS and UT, respectively. We assume that the BSs are grouped in sets of *K* elements that are linked through a limited-capacity backhaul network and the quantized version of the CSI associated with the different links between each BS and each UT is shared to all BSs, as shown in Figure 1. Without loss of generality, we consider a symmetric case where all BSs and UTs have *M* antennas, with *M* even, which is denoted by an (*M*, *M*, *K*) interference channel, with *d*_*k*_ = *d*, for all *k* streams per user. The results of this paper can be generalized to a network with different number of antennas as long as IA remains feasible [21]. Since each BS is allowed to transmit *d* = *M*/2 data symbols on each subcarrier, this system has *KM*/2 DoF per subcarrier. Note that, with IA strategy, the capacity for any user is half the rate of its interference-free capacity [21]. An OFDM modulation with *N* available subcarriers is employed at each BS and linear precoding is done separately by each of the *N* subcarriers.

Under linear precoding, the received frequency-domain signal (i.e., after cyclic prefix removal and discrete Fourier transform (DFT) operation) at the *k*th UT and the *l*th subcarrier (*l* = 0,…, *N* − 1) is given by

(1) $$\begin{array}{c} y_{k,l}=H_{k,k,l}W_{k,l}s_{k,l}+\overset{K}{\underset{\begin{array}{c} j=1\\ j\neq k \end{array}}{\sum }}H_{k,j,l}W_{j,l}s_{j,l}+n_{k,l}, \end{array}$$

provided that the cyclic prefix is long enough to account for different overall CIRs between the BSs and the UTs (i.e., including transmit and receive filters, multipath propagation effects, and differences in the time-of-arrival for different BS-to-UT links). **s**_*k*,*l*_ is the data symbols vector of size *M*/2 × 1, with E[**s**_*k*,*l*_ **s**_*k*,*l*_^*H*^] = **I**_*M*/2_; **W**_*j*,*l*_ ∈ *ℂ* ^*MxM*/2^ is the linear precoding matrix computed at BS *j* on subcarrier *l*, normalized such that ||**W**_*j*,*l*_||_*F*_^2^ = *P*_*t*_ and *P*_*t*_ is the transmit power at the BSs; and $H_{k,j,l}^{}=\sqrt{\rho_{k,j}}H_{k,j,l}^{\left(iid\right)}$ is a size-*M* × *M* matrix with the overall channel between the *j*th BS and the *k*th UT on the *l*th subcarrier. **H**_*k*,*j*,*l*_^(*iid*)^ contains the fast fading coefficients with i.i.d. *𝒞𝒩*(0,1) entries (independent, identically distributed complex normal random variables) and *ρ*_*k*,*j*_ = 2*σ*_*k*,*j*_^2^ represents the long term channel power on the same link. **n**_*k*,*l*_ is the additive white Gaussian noise (AWGN) vector at UT *k* on subcarrier *l*, that is, **n**_*k*,*l*_ ~ *𝒞𝒩*(0, *σ*_*n*_^2^ **I**_*M*_).

The soft estimated symbols associated with the user *k* on subcarrier *l* are given by

(2) s^k,l=Gk,lHk,k,lWk,lsk,l +∑j=1j≠kKHk,j,lWj,lsj,l+Gk,lnk,l,

where **G**_*k*,*l*_ denotes the linear receiving filter employed at the UT *k* on subcarrier *l*, with dimension of *M*/2 × *M*.

## 3. Channel Quantization Strategies

### 3.1. RVQ Strategy

We briefly describe the RVQ CSI feedback quantization technique often considered for IA-based systems, which is used here for comparison purposes [19, 22]. For the sake of simplicity, we will drop the dependence on both transmit and receive antenna indexes. Thus, the CFR, between a given transmit-receive antenna link, is denoted by **h** = [*h*_0_ *h*_1_ ⋯ *h*_*N*−1_]^*T*^. The constructed codebook for channel direction information (CDI), defined as the normalized CSI (i.e., **h**_*k*_^*d*^ = **h**_*k*_/|**h**_*k*_|), is formed by 2^*B*^ vectors i.i.d. on the *M*-dimensional unit sphere, {**c**_*b*_}, *b* = 1,…, 2^*B*^, where *B* represents the number of feedback bits. Each user quantizes its CDI to a codeword in a given codebook **C**_*k*_ ∈ *ℂ* ^*M*×2^*B*^^ and the codebook is predetermined and known at both the BSs and user sides. Partial CSI is acquired at the transmitter via a finite-rate feedback channel from each of the receivers. Furthermore, we use the minimum Euclidean distance to choose the codeword closest to each channel vector direction; that is,

(3) $$\begin{array}{c} F_{i,j,m}=\arg\underset{b=1,\ldots ,2^{B_{1}}}{\min}{||h_{i,j,m}^{d}-c_{b}||}^{2}, \end{array}$$

with *i*, *j* = 1,…, *K*, *m* = 1,…, *M*. Thus, after the UT having sent the index of the codeword to the BS, it provides the channel used to design the precoder matrices, given by **h**_*i*,*j*,*m*_^*Q*^ = **c**_*F*_*i*,*j*,*m*__ [16]. The number of quantization bits for the OFDM system is given by *BMK* ^2^ *N*.

### 3.2. Proposed Sample-by-Sample Strategies

In this subsection we describe the proposed efficient channel quantization procedures, where a quantized version of the CSI associated with the different links between BSs and UTs is fed back from the UT to the BS, which sends it to the other BSs through a limited-capacity backhaul network. This information is then used by each BS to perform the overall IA design. We consider that CIR is represented by $\tilde{h}\left(\Delta T_{i}\right)$ for OFDM symbol *i*, with Δ*T*_*i*_ = *iT*_*s*_, *i* ∈ *ℕ*, or in a simplified form by $\tilde{h}={\left[{\tilde{h}}_{0}\;{\tilde{h}}_{1}\;\cdots \;{\tilde{h}}_{N-1}\right]}^{T}=F^{-1}h$, where **F** denotes an appropriate DFT matrix, with each component given by ${\tilde{h}}_{l}\left(\Delta T_{i}\right)={\tilde{h}}_{BB}\left(l\Delta t_{s}+\Delta T_{i}\right)$, for *l* = 1,…, *N* − 1; Δ*t*_*s*_ = *T*_*s*_/*N*, *T*_*s*_ is the duration of the OFDM block, ${\tilde{h}}_{BB}\left(t\right)=\sum_{j=0}^{L-1}\beta_{j}\delta \left(t-\tau_{j}\right)$ is the complex baseband representation of the CIR, *L* is the number of paths, *β*_*j*_ is the complex amplitude of the *j*th path, and *τ*_*j*_ is the delay of the *j*th path. Of these *N* CIR components, just *L* are non-zero. Let us define Ψ as the group with the position of the nonzero CIR components in $\tilde{h}$ of size *L*.

The CFR is estimated at the receiver through appropriate training sequences and/or pilots. Assuming severely time-dispersive channels, the RVQ-based schemes require the quantization of *N* samples. To reduce it, we propose a new method which only requires part of CFR. In order to further reduce the overhead for CSI quantization, some new strategies are proposed based on part of CIR quantization, which are next described in detail: CIR-quadrature and in-phase (CIR-QI), CIR-amplitude and phase (CIR-AP), CIR-phase (CIR-P), and CIR-Doppler (CIR-D) quantization strategies.  Table 1 presents the number of quantization bits required for each of the quantization strategies for a single OFDM block and for a set of *I* blocks, including the training sequence.

#### 3.2.1. CFR Quantization

We propose a new method which only requires the quantization of part of CFR. The CIR has a duration (notice that the referred duration is measured in terms of number of samples) that must be smaller than the duration of the cyclic prefix, *N*_CP_, which for typical OFDM implementations is much lower than *N*. Therefore, we have ${\tilde{h}}_{n}=0$ for *n* > *N*_CP_; that is, only the first *N*_CP_ samples of the CIR are non-zero (it should be pointed out that the samples ${\tilde{h}}_{n}$ are not necessarily associated to a given multipath component when the multipath components are not symbol-spaced). Therefore, when *N* ≥ *N*_CP_ it is enough to sample the CFR at a rate *N*/*N*_CP_; that is, we only need *N*_CP_ equally spaced samples of the CFR to obtain it without loss of information [23]. Thus, we only need to quantize *N*_CP_ equally spaced samples of **h** (i.e., we quantize the samples *h*_*l*′_ with *l*′ = 0, *N*/*N*_CP_, 2*N*/*N*_CP_,…). This is formally equivalent to quantize and send the first *N*_CP_ samples of $\tilde{h}$. We consider the separate quantization of the real and imaginary parts of each of the appropriate *N*_CP_ samples of **h**, leading to

(4) hl′Q=fQ(Re{hl′})+ιfQ(Im⁡{hl′}),

where *f*_*Q*_(·) denotes the quantization characteristic. This means that we only need 2*mN*_CP_ ≪ 2*mN* bits to send the channel information from the UTs to the BSs (and this must be done for each link between transmit and receive antenna; that is, the number of bits is 2*mN*_CP_ *M* ^2^ *K* ^2^).

#### 3.2.2. CIR-QI Quantization

In order to optimize the number of bits needed to quantize the CSI and since there are only *L* (with *L* ≪ *N*_CP_ and *N*_CP_ ≪ *N*) non-zero components in CIR, we can just quantize these *L* components (the delays and their complex amplitude). The delays just need to be transmitted once, since they are usually constant for over a large number of data blocks. Therefore, we quantize ${\tilde{h}}_{p}\left(\Delta T_{i}\right)=\beta_{p}\left(\Delta T_{i}\right)$, with *p* ∈ Ψ, and *β*_*p*_(Δ*T*_*i*_) ∈ *ℂ*, which corresponds to the *p*th non-zero component of the *i*th OFDM block. We consider the separate quantization of the real and imaginary parts of each component of $\tilde{h}\left(\Delta T_{i}\right)$, leading to

(5) h~pQ(ΔTi)=fQ(Re{h~p(ΔTi)})+ιfQ(Im⁡{h~p(ΔTi)}).

The number of quantization bits required is only 2*mLM* ^2^ *K* ^2^ per OFDM block, with *m* denoting the number of bits required for the real and imaginary parts of each quantized component, which is much lower than in the case of CFR quantization strategy.

#### 3.2.3. CIR-AP Quantization

Another quantization strategy is based on the previous one, consisting in quantizing the amplitude and phase of the non-zero components of CIR, separately, so that

(6) $$\begin{array}{c} {\tilde{h}}_{p}^{Q}\left(\Delta T_{i}\right)=f_{Q}\left(\left|{\tilde{h}}_{p}\left(\Delta T_{i}\right)\right|\right)e^{\iota f_{Q}(\arg({\tilde{h}}_{p}(\Delta T_{i})))}. \end{array}$$

Again, the number of quantization bits required is only 2*mLM* ^2^ *K* ^2^ per OFDM symbol.

#### 3.2.4. CIR-P Quantization

A fourth approach is presented for the case of slow varying channels, where the CIR is constant over a set of OFDM blocks; that is, ${\tilde{h}}_{p}\left(\Delta T_{i}\right)=\left|{\tilde{h}}_{p}\left(0\right)\right|e^{\iota \arg({\tilde{h}}_{p}(\Delta T_{i}))}$. In this case, for each set of OFDM blocks, we just need to quantize the amplitude, phase, and delays for the first OFDM symbol. Then, for the following symbols, only the phase needs to be quantized, reducing the overhead to one half of the overhead of the previous strategy (i.e., *mLM* ^2^ *K* ^2^ bits per symbol).

#### 3.2.5. CIR-D Quantization

In this case, we consider that there are small variations in the phase of each channel delay path with time, due to same scattering in reflections. Thus, in this case each CIR component for block *i* is given by ${\tilde{h}}_{p}\left(\Delta T_{i}\right)={\tilde{h}}_{p}\left(0\right)e^{\iota 2\pi f_{D}\cos(\theta_{p}(\Delta T_{i}))\Delta T_{i}}$, where *θ*_*n*_ is the angle of arrival (AoA) and follows a uniform distribution between 0 and 2*π*. Assuming small differences in the AoA, then the *p*th channel component can be approximated as

(7) $$\begin{array}{c} {\tilde{h}}_{p}\left(\Delta T_{i}\right)={\tilde{h}}_{p}\left(0\right)e^{\iota 2\pi D_{p}\Delta T_{i}}, \end{array}$$

where *D*_*p*_ is the Doppler term for the first symbol; that is, *D*_*p*_ = *f*_*D*_cos⁡(*θ*_*p*_(0)). In this case, we can quantize the delays, the amplitude, and the phase of each path and also the Doppler effect term, just for the first block. In this strategy, we must make sure that *f*_*D*_ *T*_OFDM_ ≪ 1, so that the channel is invariant in an OFDM block duration. For the remaining set of *I* blocks, the CIR components of *i*th block, with *i* = 1,…, *I*, can be estimated according to

(8) $$\begin{array}{c} {\tilde{h}}_{p}^{Q}\left(\Delta T_{i}\right)=f_{Q}\left(\left|{\tilde{h}}_{p}\left(0\right)\right|\right)e^{\iota f_{Q}(\arg({{\tilde{h}}_{p}}^{}(0)))+\iota 2\pi f_{Q}(D_{p})\Delta T_{i}}. \end{array}$$

The number of bits is just 4*mLM* ^2^ *K* ^2^ for a set of *I* OFDM blocks.

#### 3.2.6. Power of the Quantization Error

In this subsection, we present an analytical approach to compute the power of the noise variance. Throughout this paper, we assume that each CIR component is approximately Gaussian. According to the Central Limit Theorem, the average of a sufficiently large number of iterates of i.i.d. random variables is approximately normally distributed [24]. Thus, modeling each CIR component as Gaussian is a reasonable approximation when we have reflections on irregular surfaces, since each CIR component can be regarded as a sum of several rays that arrive approximately with the same delay and AoA. In this case, it can be shown that the quantized components of the CIR are approximately given by (see [25])

(9) $$\begin{array}{c} {\tilde{h}}_{p}^{Q}\approx \alpha_{p}{\tilde{h}}_{p}+n_{p}^{Q},\;p\in \Psi , \end{array}$$

with *n*_*p*_^*Q*^ denoting the quantization noise term with variance *σ*_*Q*_^2^. As *n*_*p*_^*Q*^ and ${\tilde{h}}_{p}$ are uncorrelated, we can obtain the *α*_*p*_ factor as

(10) αp=E[h~pQ∗h~p]E[|h~p|2]=12πσp3∫−∞+∞h~pfQ(h~p)e−(h~p)2/2σp2dh~p,

with the useful power for multipath *p* is given by $P_{u,p}=2\sigma_{p}^{2}=\text{E}\left[{\left|{\tilde{h}}_{p}\right|}^{2}\right]$. The average power of quantization noise term, *P*_*ε*,*p*_, is expressed through [25]

(11) $$\begin{array}{c} P_{\varepsilon ,p}=\frac{1}{\sqrt{2\pi }\sigma_{p}}\overset{+\infty }{\underset{-\infty }{\int }}f_{Q}^{2}\left({\tilde{h}}_{p}\right)e^{-{\left({\tilde{h}}_{p}\right)}^{2}/2\sigma_{p}^{2}}d{\tilde{h}}_{p}-{\left|\alpha \right|}^{2}\sigma_{p}^{2}. \end{array}$$

The signal-to-quantization noise ratio (SQNR) on subcarrier *p* is given by

(12) $$\begin{array}{c} \text{SQN}{\text{R}}_{p}=\frac{P_{t,p}}{P_{\varepsilon ,p}} \end{array}$$

with the total signal power obtained by *P*_*t*,*p*_ = *α*_*p*_^2^ *P*_*u*,*p*_ + *P*_*ε*,*p*_. As in this paper we consider uniform quantizers with 2^*m*^ levels and normalized saturation level *A*_*M*_/*σ*, we therefore can write the integrals of (10) and (11) in a closed form as sums.

## 4. IA Algorithms

In this section, we start by briefly presenting the closed-form IA-MMSE algorithm for *K* ≤ 3. Then, the iterative MMSE-based IA algorithm for a general *K* and assuming perfect CSI is described. After that, the MMSE is explicitly minimized under channel quantization errors, referred to as IA robust IMMSE algorithm.

### 4.1. Closed-Form IA Precoder for *K* = 3

For the three-user interference channel, it is possible to find a closed-form solution to precoding matrices **W**_*k*,*l*_, *k* = 1,2, 3, although not necessarily the best solution for low-to-moderate SNR values. As shown in [1], the solution for subcarrier *l* is given by

(13) W1,l=[ω1ω2…ωM/2],W2,l=H3,2,l−1H3,1,lW1,l,W3,l=H2,3,l−1H2,1,lW1,l,

where **ω**_1_, **ω**_2_,…, **ω**_*M*/2_ are the eigenvectors of matrix **ω**_*l*_ = **H**_3,1,*l*_^−1^ **H**_3,2,*l*_ **H**_1,2,*l*_^−1^ **H**_1,3,*l*_ **H**_2,3,*l*_^−1^ **H**_2,1,*l*_.

At the receiver, we employ an MMSE equalizer to separate the desired spatial streams. The equalizer matrix can be written as

(14) $$\begin{array}{c} {\overset{-}{G}}_{k,l}={\left(H_{\text{eq},k,l}^{H}H_{\text{eq},k,l}+\sigma_{n}^{2}I_{3M/2}\right)}^{-1}H_{\text{eq},k,l}^{H}, \end{array}$$

where $H_{\text{eq},k,l}=\left[H_{k,1,l}{\overset{-}{W}}_{1,l}\;H_{k,2,l}{\overset{-}{W}}_{2,l}\;H_{k,3,l}{\overset{-}{W}}_{3,l}\right]$ is of size *M* × 3*M*/2. From (9), the linear filter used at the *k*th receiver is given by

(15) Gk,l=[gk,(k−1)M/2+1,lTgk,(k−1)M/2+2,lT⋯gk,(k−1)M/2+M/2,lT]T

with dimension (*M*/2 × *M*) and **g**_*k*,*j*,*l*_ is the *j*th row vector of ${\overset{-}{G}}_{k,l}$.

### 4.2. Iterative MMSE IA Algorithm

A promising iterative MMSE (IMMSE) IA approach for a generic *K* was proposed in [8, 9]. The MMSE criterion minimizes the expected sum of the norms between each ${\hat{s}}_{k,l}$ and **s**_*k*,*l*_ given by

(16) $$\begin{array}{c} {ℑ}_{\text{MSE}}=\overset{K}{\underset{k=1}{\sum }}\text{E}\left\{{||{\hat{s}}_{k,l}-s_{k,l}||}^{2}\right\}=\overset{K}{\underset{k=1}{\sum }}\text{E}\left\{{||G_{k,l}y_{k,l}-s_{k,l}||}^{2}\right\}, \end{array}$$

and the optimization problem can be formulated as

(17) min⁡ℑMSE({Wj,l},{Gk,l}),s.t.||Wj,l||2=Pt, j∈{1,…,K}.

The solution is derived through the Karush-Kuhn-Tucker (KKT) conditions

(18) ∂L(Wj,l,Gk,l,λj)∂Wj,l=0,∂L(Wj,l,Gk,l,λj)∂Gk,l=0,∂L(Wj,l,Gk,l,λj)∂λj=0, k,j=1,…,K,

where *λ*_*j*_ is the Lagrange multiplier associated with the power constraint of transmitter *j* and the Lagrangian function is given by

(19) $$\begin{array}{c} L\left(W_{j,l},G_{k,l},\lambda_{j}\right)={ℑ}_{\text{MSE}}+\overset{K}{\underset{j=1}{\sum }}\lambda_{j}\left(\mathrm{tr}\left(W_{j,l}W_{j,l}^{H}\right)-P_{t}\right). \end{array}$$

The optimum solution is given in the following iterative procedure:

(1) fix **W**_*j*,*l*_ arbitrarily for all *j* on each *l*;
(2) calculate matrix **G**_*k*,*l*_ given by
  (20) $$\begin{array}{c} G_{k,l}=W_{k,l}^{H}H_{k,k,l}^{H}{\left(\overset{K}{\underset{j=1}{\sum }}H_{k,j,l}W_{j,l}W_{j,l}^{H}H_{k,j,l}^{H}+\sigma_{n}^{2}I_{M}\right)}^{-1}; \end{array}$$
(3) find *λ*_*j*_ that solves tr⁡(**W**_*j*,*l*_^*H*^ **W**_*j*,*l*_) = *P*_*t*_ for *j* = 1,2,…, *K*, with **W**_*j*,*l*_ given by
  (21) $$\begin{array}{c} W_{j,l}={\left(\overset{K}{\underset{k=1}{\sum }}H_{k,j,l}G_{k,l}G_{k,l}^{H}H_{k,j,l}^{H}+\lambda_{j}I_{M}\right)}^{-1}H_{j,j,l}^{H}G_{j,l}^{H}; \end{array}$$
(4) update **W**_*j*,*l*_ and **G**_*k*,*l*_ with the obtained *λ*_*j*_;
(5) repeat steps (2) to (4) until convergence or a predefined number of iterations is reached.

### 4.3. Robust IMMSE IA Algorithm

As discussed, in practical scenarios it is not realistic to consider perfect CSI. The channel errors due to channel estimation and/or quantization may have significant impact on the IA algorithm performances, mainly for high SNRs (this effect can be observed in Section 5, when the BER curves do not always decrease with SNR). We propose a robust IA IMMSE algorithm, where the MMSE is explicitly minimized by considering the channel quantization errors. The overall channel frequency domain matrix, when taking into account the channel quantization errors, can be modeled as **H**_*i*,*j*,*l*_^*Q*^ = **H**_*i*,*j*,*l*_ + **E**__*i*,*j*,*l*__^*Q*^, *i*, *j* = 1,…, *K*, where **H**_*i*,*j*,*l*_^*Q*^ represents the overall quantized channel matrix and **E**_*i*,*j*,*l*_^*Q*^ is the overall quantized error matrix. By replacing **H**_*i*,*j*,*l*_ by **H**_*i*,*j*,*l*_^*Q*^ − **E**_*i*,*j*,*l*_^*Q*^ in (17), we obtain the MSE when we have errors in the CIR (due to quantization effects and/or CIR estimation errors), given by

(22) ℑMSE=∑k=1K(tr⁡(E[∑j=1KGk,l(Hk,j,lQ−Ek,j,lQ)Wj,lWj,lH          ×(Hk,j,lQ−Ek,j,lQ)HGk,lH])     −2tr⁡(E[Gk,l(Hk,k,lQ−Ek,k,lQ)Wk,l])     +σn2tr⁡(Gk,lGk,lH)+M2).

This expression can then be used to solve (17), whose solution, which can once again be derived through the KKT conditions, leads to the equalizer and the precoder matrices given by

(23) Gk,l=Wk,lHHk,k,lQH(∑j=1KHk,j,lQWj,lWj,lHHk,j,lQH+σn2IM          + σQ2tr⁡∑j=1K(Wj,lWj,lH)IM)−1,

(24) Wj,l=(∑k=1KHk,j,lQGk,lGk,lHHk,j,lQH     + λjIM+σQ2tr⁡(∑k=1KGk,lGk,lH)IM)−1Hj,j,lQHGj,lH,

where *σ*_*Q*_^2^ denotes the variance of the channel error. Naturally, when this variance tends to zero, (23) and (24) tend to the conventional equalizer and precoder matrices, respectively.

The iterative procedure is identical to the conventional IA-MMSE based algorithms, where in this case the precoder and equalizer matrices are replaced by (23) and (24), respectively.

## 5. Performance Results

In this section, we present a set of performance results for the IA techniques described above under the proposed channel quantization schemes, namely, the closed-form MMSE approach (MMSE), the iterative MMSE (I-MMSE) and the proposed robust IMMSE (R-IMMSE). We evaluate the four proposed quantization strategies and the case of perfect knowledge of the CSI (P-CSI) at the BSs. For the sake of comparison, we also evaluate the strategy with feedback quantization of CDI using RVQ. We consider error-free feedback links and we assume that the CSI is perfectly estimated at the UTs.

Our scenario has *K* = 3 BSs, cooperating to transmit information to *K* = 3 UTs sharing the same resources. All terminals are equipped with 4 antennas. The main parameters used in the simulations are based on LTE standard [26]: FFT size of 1024; cyclic prefix of 64; sampling frequency set to 15.36 MHz; subcarrier separation is 15 kHz, subcarrier frequency is 2 GHz, the OFDM symbol duration is 66.7 *μ*s, and the modulation is QPSK. For the Doppler quantization strategy we consider 120 km/h as the maximum velocity, which corresponds to a maximum normalized Doppler frequency of *f*_*D*_ *T*_*s*_ = 0.0148. The number of iterations used in simulations of the IMMSE algorithm is 20, since we observe that the gains obtained with more than this number of iterations is negligible (this number was also used in other works such as [9, 20]). We adopted the pedestrian ITU BRAN B channel, with 6 paths in the power delay profile according to [27]. We assume perfect CSI estimation at receiver side (i.e., at the UT). We consider uniform quantizers with 2^*m*^ levels and normalized saturation level *A*_*M*_/*σ*, with 2*σ* ^2^ = *E*[|*h*_*l*_|^2^], with *l* = 1,…, *N*.

**Figure 2:**
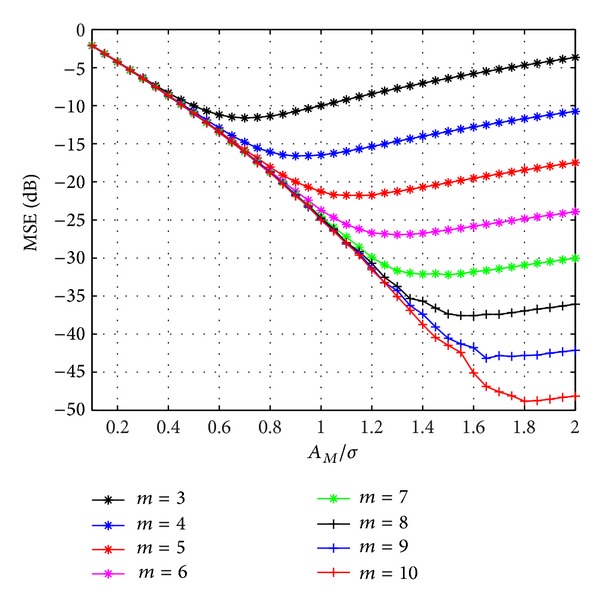
MSE of CIR quantization in function of clipping value, for different number of quantization bits.

Figure 2 shows the impact of the normalized saturation level *A*_*M*_/*σ* and the number of quantization *m* on SQNR. As can be observed, there is an optimum normalized saturation level for each value of *m*, since the quantizer's saturation becomes too frequent if *A*_*M*_/*σ* is small and the quantization interval becomes too high when *A*_*M*_/*σ* is high. Hereinafter, we assume always the optimum saturation level for each value of *m* in the proposed quantization schemes.

Performances of IA algorithms are presented in terms of the average bit error rate (BER) as a function of *E*_*b*_/*N*_0_, with *E*_*b*_ denoting the average bit energy and *N*_0_ denoting the one-sided noise power spectral density.

**Figure 3:**
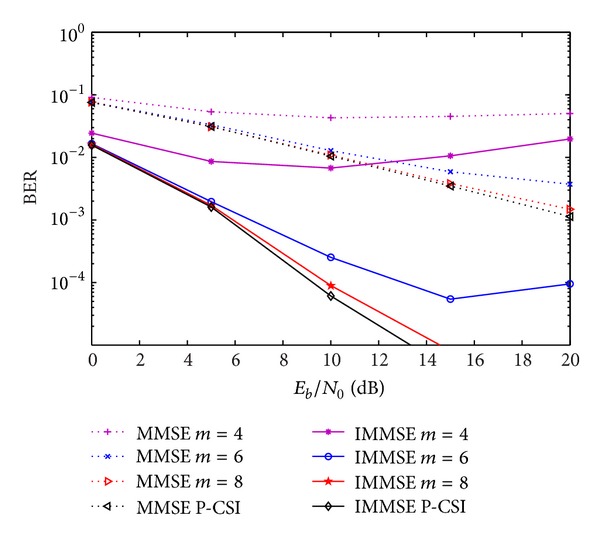
Performance of IA MMSE and IMMSE algorithms with CIR-QI quantization, for different number of quantization bits.

**Figure 4:**
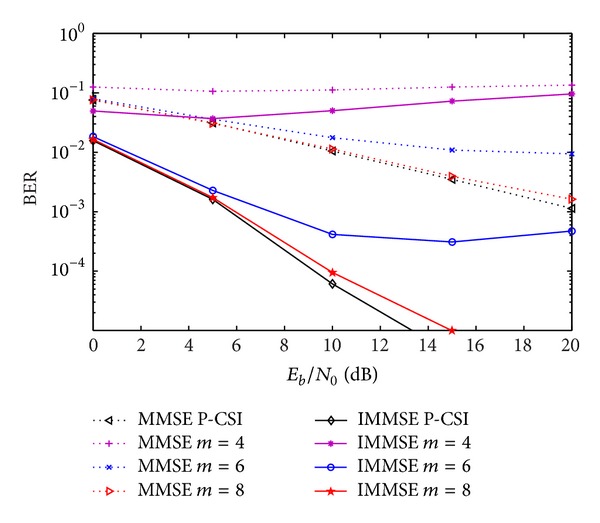
Performance of IA MMSE and IMMSE algorithms with CIR-AP quantization, for different number of quantization bits.

**Figure 5:**
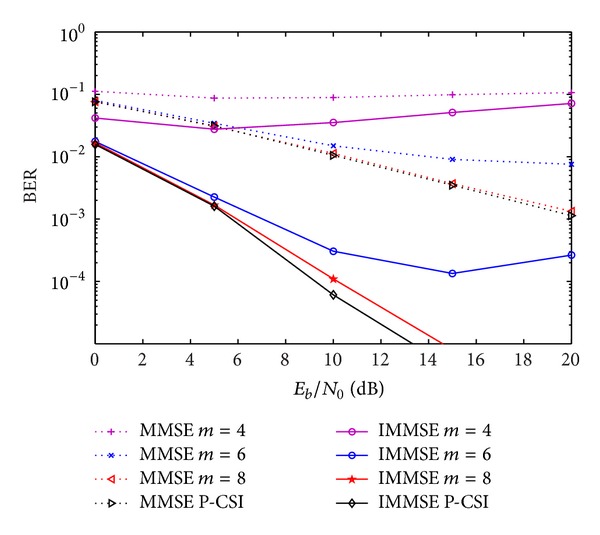
Performance of IA MMSE and IMMSE algorithms with CIR-P quantization, for different number of quantization bits.

**Figure 6:**
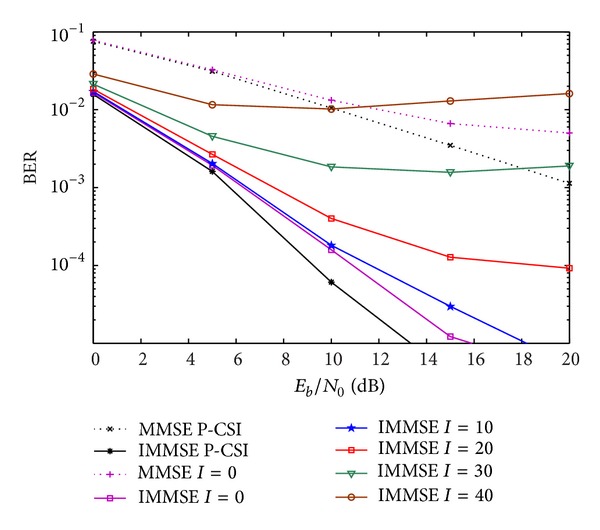
Performance of IA MMSE and proposed IMMSE algorithms, with CIR-D, for *m* = 6 bits.

**Figure 7:**
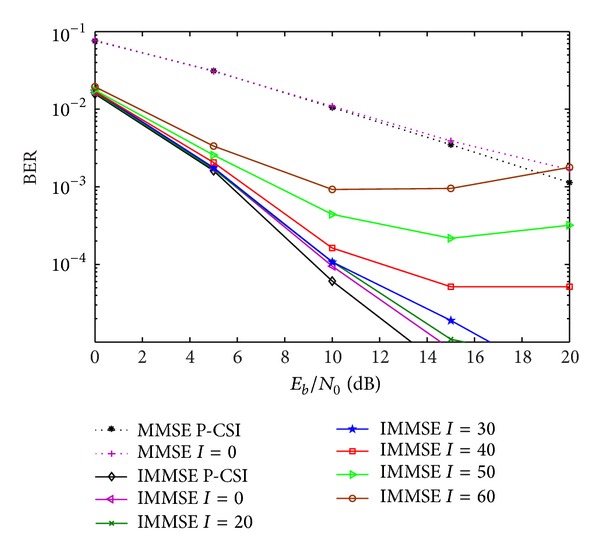
Performance of IA MMSE and proposed-IMMSE algorithms with CIR-D quantization, for *m* = 8 bits.

Figures 3–7 show the impact of quantization on the BER performance for *m* equals 4, 6, and 8 bits and for perfect knowledge of CSI (no CSI quantization). The results are presented for the closed-form MMSE and IMMSE approaches. We considered the four approaches for CIR quantization, presented in Section 3. In the case of quantization of real and imaginary parts of the non-zero components of CIR (CIR-QI), the performances are shown in Figure 3. As expected, the quantization degrades the system's performance. Increasing the number of quantization bits, the performance of the IA algorithms tends to the ones obtained with perfect CSI. For *m* = 6 the performance penalty is not significant for low and medium values of *E*_*b*_/*N*_0_. For *m* = 8, we can see that the performance is very close to the one obtained for perfect CSI.

Figure 4 depicts the BER performance comparisons for the same schemes, but considering the CIR quantization in terms of amplitude and phase (CIR-AP). The IA algorithms present a slightly higher degradation than the previous one, mainly for low number of quantization bits, since the quantization characteristic amplitude is larger for the quantization of the phase (from 0 to 2*π*) than for the real and imaginary components of the CIR samples.

In Figure 5, the plots for the same schemes with the third quantization approach (CIR-P) are presented. In this case, the amplitude for of each non-zero CIR component is just transmitted once for a group of OFDM blocks, reducing the number of bits to approximately one-half of the last case. We observe a slight improvement comparative to the previous case, since this strategy is used in cases of slow-varying channels, thus having almost constant amplitude taps.

Figures 6 and 7 show the performance of the IA algorithms with the CIR-D quantization for *m* = 6 and 8 bits, respectively. In this case, the delays amplitude and phase, and the Doppler effect parameters are quantized just for the first OFDM block. For the following ones, an estimation is made according to the quantized parameters. We present the BER performances for a set of *I* OFDM blocks after the quantized and fed back OFDM block, with *I* = 10,20,…, 50. For the case where *m* = 6 (depicted in Figure 6), there is no significant degradation for 10 OFDM blocks without feedback of CSI. The number of bits needed in this scheme over the ones of the CFR quantization is *N*_*f*6_/*N*_*f*2_ = 2*L*/*N*_CP_ *I* and *N*_*f*6_/*N*_*f*3_≃2/*I* for CIR-QI. This allows a reduction of about 80% of the overhead comparative to the CIR-QI. It presents some penalty for more than 20 OFDM blocks without feedback, specially for high *E*_*b*_/*N*_0_ values. For the scenario represented in Figure 7, we have performances close to the case of perfect CSI for a long set of OFDM blocks, with almost no overhead. For example the CSI is known in BSs for a set of 30 OFDM symbols with just a penalty of 1 dB (for a BER of 10^−4^), only using 0.6% of the quantization bits used in the IA conventional case with CFR where all subcarriers of the channel are quantized (i.e., *N*_*f*6_/*N*_*f*2_).

**Figure 8:**
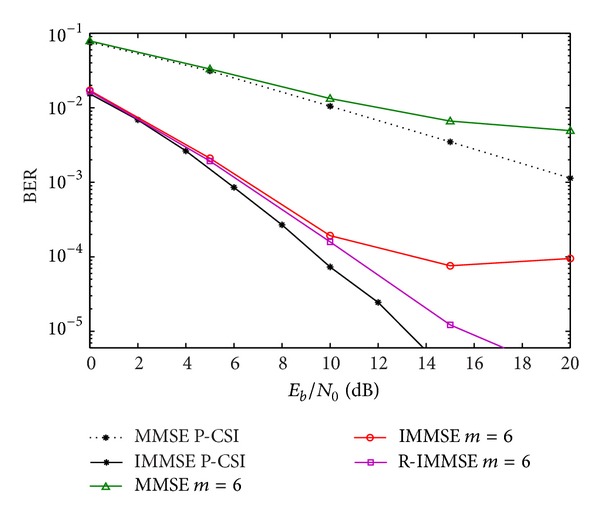
Performance of IA MMSE, IMMSE, and proposed R-IMMSE algorithms with CFR quantization, for *m* = 6 bits.

Figure 8 plots the performance of the MMSE, IMMSE, and the proposed R-IMMSE with the CFR feedback strategy, with *m* = 6 bits. We observe that the robust algorithm clearly outperforms the nonrobust iterative one, since the robust one is designed taking into account the quantization errors. Also we can observe that the performance gains are higher for medium to high SNR regime, since this is the region where the quantization errors strongly affect the systems performance.

**Figure 9:**
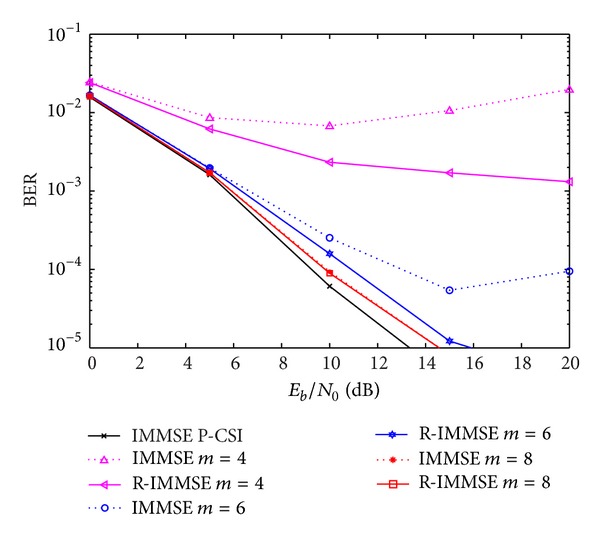
Performance of IA MMSE and R-IMMSE algorithms with CFR quantization, for different number of quantization bits.

In Figure 9 we compare the performance of the proposed robust with the non-robust IA MMSE algorithms, for different values of *m*. From the figure we can observe that the robust algorithm clearly outperforms the conventional one for low to moderate number of quantization bits, and mainly for moderate to high SNR regimes. As expected, when the number of quantization bits increases the robust scheme tends to the conventional one, since the quantization error power tends to zero. For *m* = 8, we can see that the performance of both algorithms is very close to the one obtained for perfect CSI, with less than 0.5 dB of difference for BER = 10^−4^.

**Figure 10:**
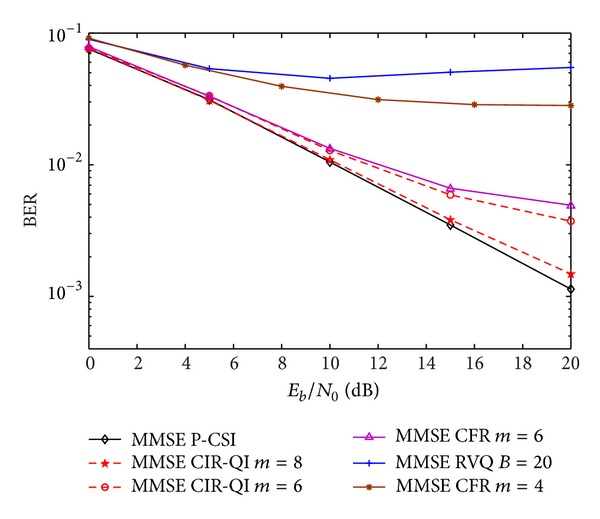
Performance of IA MMSE algorithm with CIR-QI, CFR, and RVQ quantizations.

In Figure 10, the RVQ strategy is presented and compared with the proposed CIR-QI quantization. We observe a large penalty in using RVQ with 20 bits per user per antenna. As expected from the analysis in other works using RVQ quantization, the capacity saturates at a constant value if the number of feedback bits is fixed. In [17], the authors improve the multiplexing gain of the proposed scheme by increasing the number of feedback bits scaling with SNR. However, the simulations with more than 20 bits for the RVQ strategy results in a higher computational effort and increases even more the number of feedback overhead. Notice that the overhead rate between CIR-QI and RVQ quantizations is given by *N*_*f*3_/*N*_*f*1_≃2*mML*/*BN* bits, which is equivalent to has only 15% of the required feedback overhead with the proposed quantization than with the RVQ, for the simulated system with *m* = 6 and *B* = 20, for example. Comparing the RVQ with the CIR-D, the reduction obtained is 1 − *N*_*f*6_/*N*_*f*1_≃1 − 4*mML*/*BN* *I*, which is about 99.7% for the same parameters and 10 OFDM blocks. In the case of more quantization bits being used for RVQ case, our proposal becomes even more advantageous.

## 6. Conclusions

In this paper, several channel quantization strategies for IA MMSE-multicell-based systems were proposed. The quantization methods require low-complexity and low-bit rates, and imply quantization of different channel parameters: just part of the channel frequency response or only the CIR's non-zero taps. The considered schemes were studied in detail and evaluated under practical scenarios based on LTE parameters. The proposed algorithms outperform the RVQ-based ones with a large reduction in the required overhead. Moreover, the effect of CSI quantization errors is almost negligible for a reduced number of quantization bits. We also observed the effect of just transmitting the main CSI parameters for one OFDM block and estimated the CSI for the following blocks, drastically reducing the amount of data needed (even when compared with the other proposed strategies with low overhead), presenting lower penalties when comparing with the cases where the CSI is always obtained for each block.

To overcome the penalty introduced by the channel errors due to CSI quantization/estimation in the IA algorithms, we also proposed a robust iterative IA MMSE-based algorithm, where quantization errors are taken into account in IA design. This algorithm was also evaluated under the proposed limited feedback strategies and clearly outperformed the conventional iterative IA MMSE algorithm, mainly for medium and high SNR.

The proposed techniques are of great importance to practical systems, where it is impossible to avoid CSI errors, showing to have performances close to the ones with perfect knowledge of CSI and with lower complexity and overhead than the RVQ-based approaches.

## Figures and Tables

**Table 1:** Number of quantization bits required for the discussed quantization strategies for one OFDM block and for one frame (set of *I* blocks and a training sequence).

Quantization strategy	Number of quantization bits per block	Number of quantization bits per frame
RVQ	*N*_*b*1_ = *BMK* ^2^ *N*	*N*_*f*1_ = *BMK* ^2^ *NI*
CFR	*N*_*b*2_ = 2*mN*_CP_ *M* ^2^ *K* ^2^	*N*_*f*2_ = 2*mN*_CP_ *M* ^2^ *K* ^2^ *I*
CIR-QI	*N*_*b*3_ = 2*mLM* ^2^ *K* ^2^	*N*_*f*3_ = *mLM* ^2^ *K* ^2^(2*I* + 1)
CIR-AP	*N*_*b*4_ = 2*mLM* ^2^ *K* ^2^	*N*_*f*4_ = *mLM* ^2^ *K* ^2^(2*I* + 1)
CIR-P	*N*_*b*5_ = *mLM* ^2^ *K* ^2^	*N*_*f*5_ = *mLM* ^2^ *K* ^2^(*I* + 2)
CIR-D	—	*N*_*f*6_ = 4*mLM* ^2^ *K* ^2^
